# Perioperative antibiotic prophylaxis in the setting of cochlear implantation: a retrospective analysis of 700 cases

**DOI:** 10.1007/s00405-024-08515-1

**Published:** 2024-03-14

**Authors:** Katharina Schaumann, D. Mütz, L. Althaus, T. Prinzen, J. Schipper, T. Klenzner

**Affiliations:** Department of Otorhinolaryngology, Medical Faculty and University Hospital Düsseldorf, Heinrich-Heine-University Düsseldorf, Moorenstraße 5, 40255 Düsseldorf, Germany

**Keywords:** Cochlear implantation, Revision surgery, Perioperative antibiotic prophylaxis, Surgical site infection, Wound infection, Explantation

## Abstract

**Purpose:**

Postoperative wound infections after cochlear implantation are rare but sometimes serious and can lead to explantation. Therefore, perioperative antibiotic administration is often recommended. However, in clinical practice, the type and duration of antibiotic prophylaxis varies between different centers. The aim of this study was to investigate the role of perioperative antibiotic prophylaxis in preventing postoperative complications.

**Methods:**

700 patients who underwent cochlear implantation between 2007 and 2019 were retrospectively evaluated with regard to wound infections within the first 28 postoperative days. These were classified into major and minor complications. Data were analyzed using the IBM statistical program SPSS.

**Results:**

In 670 out of 700 patients the type and duration of perioperative antibiotic administration could be reconstructed from the records. Of these 67 patients (10%) received antibiotics as a single shot, 158 patients (23.6%) were treated with antibiotics for a period of 48 h, and 445 patients (66.4%) received prolonged antibiotic therapy for more than 72 h. In total 64 patients (9.5%) showed abnormalities in wound assessment within the first 28 postoperative days after implantation. Major infections (1.6%) were detected in 11 patients. Overall, there was no statistically significant difference in wound infection rates between the group receiving single-shot antibiosis and the group receiving 48 h prophylaxis or antibiotic treatment > 72 h (*p* = 0.46).

**Conclusion:**

Patients receiving an antibiotic single shot do not appear to be at significantly increased risk for postoperative wound infections compared with patients with prolonged antibiotic treatment. Continuation of data collection across centers seems reasonable.

## Introduction

Cochlear implants (CI) are currently the most successful neuroprostheses. Due to improved surgical techniques and implant technology, the indication criteria have been expanded in recent years. This is why the number of patients provided with CI is steadily increasing.

CI surgery is a low-risk surgical procedure, severe complications are rare. The incidence of postoperative wound infections reported in the literature seems to vary within a range of 1–13% [1]. Even rare, these kinds of complications are feared as they can lead to delay in hearing rehabilitation, meningitis, cerebral complications and implant infections up to explantation. Requiring repeat surgery with surgical charges of > 27,000 Euros per implantation, this represents a significant cost aspect in addition to an enormous burden for the patient. To avoid the occurrence of such "serious adverse events", perioperative antibiotic administration is performed in most clinics. Also, the administration of a single shot antibiotic prophylaxis 30 min before surgery is often recommended e.g. with strong consensus in the current German AWMF guideline. Prolonged administration of antibiotics is recommended in case of additional risks [2]. This is justified by the fact that CI surgery is classified as a clean-contaminated procedure due to the physiological germ flora of the middle ear and possible pathogen transmission from the nasopharynx via the open tuba eustachii [3]. The use of implants also carries an increased risk of postoperative wound infections. In addition, the intraoperative opening of the inner ear creates the potential for infection of the associated cerebrospinal fluid space [4].

To date, there is no clear international consensus about the use of perioperative antibiotics. The type and duration of the antibiotic regime varies between centers. The results of previous studies are inconsistent. It is therefore extremely difficult to make a universally valid, evidence-based recommendation. Considering the heterogenous data, almost all studies advocate perioperative antibiotic prophylaxis with cephazolin, cefuroxime, or amoxicillin/clavulanic acid. Duration varies from a single application to several weeks of therapy. Detected pathogens responsible for wound infection are Staphylococcus aureus, less frequently Streptococcus pneumoniae and *Haemophilus influenzae*, and in some cases *Pseudomonas aeruginosa*. Infection with the latter appears to be more severe and more often resulted in explantation [5].

A total of 700 cochlear implantations were performed at University Hospital setting between 2007 and 2019. All Patients received a perioperative antibiotic prophylaxis, but the duration of the antibiotic therapy regime varied over the last 15 years. From 2007 to 2015, longer-term antibiotic therapy of > 72 h was prescribed predominantly according to the intraclinical standard at that time. Because of increasing influence of antibiotic stewardship and the rising importance of antibiotic resistance, efforts were made to reduce antibiotic use in the following years, leading to an antibiotic prophylaxis for 48 h. Since 2019, prophylaxis is mainly limited to a perioperative single shot, according to the recommendation of the current AWMF—guideline [2].

The aim of this study was to compare different therapy regimens in a retrospective quality analysis, to record complications, and to clarify the role of perioperative antibiotic prophylaxis in preventing postoperative wound infections. If there is any difference, we anticipate that the reduction of antibiotic administration during the perioperative period will result in a clinically insignificant increase in the risk of postoperative wound infections compared to prolonged administration.

## Methods

### Population/study design

In a retrospective cohort study, the electronic files of all patients who received a unilateral or bilateral CI at the ENT University Hospital between 2007 and 2019 were analyzed within the framework of a doctoral thesis. All wound infections and complications documented in the files during the first 28 postoperative days were recorded. Patient age and sex, type and duration of perioperative antibiotic used, length of hospital stay, and infection-promoting factors such as pre-existing conditions, medications, previous surgeries, intraoperative features, surgical access route to cochlea, implant and electrode type, and isolated pathogens were documented. If a postoperative wound infection occurred, the time of occurrence, type, location, and severity of infection, as well as the therapeutic consequence were recorded (Table 1).

**Table 1:** Parameters recorded

1. Number, age, sex
2. Operation:
(a) Date, side
(b) Cochlear access: (extended) round window approach, cochleostomie,
(c) Implant and electrode type
(d) Intraoperative signs of inflammation of infection
(e) Other pathologies
(f) Days of inpatient stay
3. Perioperative administration of antibiotics:
(a) Type of antibiotics
(b) Administered dose
4. Postoperative infections:
(a) Localisation, type and severity of infection, time of occurrence, laboratory signs of infection
(b) Therapy
5. Other postoperative complications
6. Preexisting conditions
7. Medication
8. Smoking
9. Regular alcohol consumption

Wound infections were divided into major and minor infections. Major complication resulted in a new or prolonged inpatient stay, new surgical interventions, explantation, or involvement of the central nervous system. Infections for which conservative outpatient therapy was sufficient were considered minor complications. Patients were excluded from the study if the above criteria could not be completely collected from the electronic record.

All described studies were performed with the approval of the local ethics committee, in accordance with national law, and in accordance with the Declaration of Helsinki of 1975 (in the current, revised version).

### Surgical technique

After small-area retroauricular shaving, local anesthesia, surgical disinfection, and sterile draping of the surgical area, a usually S- or C-shaped skin incision was made and an opposing musculoperiosteal flap was applied. After mastoidectomy, antrostomy, and posterior tympanotomy, electrode insertion was performed via a round window approach (widened if necessary) or via a cochleostomy, depending on the choice of electrode and anatomy. The implant was placed in subperiosteal pocket in the temporal region. To avoid dislocation, a bony bed was drilled prior and the implant was fixed with sutures. Subsequently, a three-layer wound closure (periosteum, subcutaneous, cutaneous) was performed.

In case of chronically infected and/or multiple pre-operated ears (e.g. presence of a radical cavity), a subtotal petrosectomy with ear canal closure as well as obliteration of the mastoid/ middle ear with abdominal fat was usually performed first. In these cases, cochlear implantation was performed in a staged procedure after approximately 3 month when wound healing was complete. Also, some implantations were performed simultaneously or after vestibular schwannoma resection.

### Follow-up

Postoperatively, a pressure wrap dressing was applied for approximately 3 days. Usually, the inpatient stay was 2 days after surgery. During this time, a daily wound check was performed. In case of non-absorbable suture material, the skin suture was removed between the 7th–10th day. A further medical check-up was performed as part of the first rehabilitation session usually 4 weeks postoperatively. Additional individual follow-up appointments were included in the analyses until postoperative day 28.

### Statistics

The data collected from the electronic files were entered into a specially programmed input mask (supported by Serrala Group GmbH, Hamburg, Germany) using a checkbox or free field. They were subsequently transferred into Excel (Microsoft Excel) and analyzed using the IBM statistical program SPSS. For continuous data, the mean was calculated. Infection rates in the group of patients who received single-shot antibiotics were compared with those in the group of patients who received prolonged antibiotic therapy of 48 h or longer than 72 h. In addition, it was compared whether risk factors such as immunosuppressive pre-existing conditions/medications or previous surgery were associated with an increased infection rate. The influence of the above-mentioned variables on the risk of infection was calculated using binary logistic regression and a Fisher`s exact test. The results were expressed as odds ratios and 95% confidence intervals (95% CI). The significance level was set at 5% (alpha = 0.05).

## Results

A total of 700 patients received cochlear implantation during the above-mentioned period. The choice of perioperative antibiotic administration could be recorded for 670 patients from the electronic files. 30 patients had to be excluded from the study because of incomplete data. These were 492 adults (mean [SD] age, 56.5 [16.4]), of whom 209 were male (42.5%) and 283 female (57.5%).

178 patients were children (mean [SD] age, 5.06 [5.38]), of whom 82 were female (46%) and 96 male (54%).

Of these 670 patients, 65 patients received simultaneous bilateral CIs, resulting in a total of 753 implants.

A total of 67 patients (10%) received antibiotics as a single shot (single shot group), 158 patients (23.6%) were treated with antibiotics for a period of 48 h (48 h group), and 445 patients (66.4%) received prolonged antibiotic therapy for a time course more than 72 h (72 h group). (Table 2) Cefuroxime was used in 303 cases (45.2%), amoxicillin/ clavulanic acid or ampicillin/sulbactam in 23 cases (3.4%), cephazolin in 113 cases (16.8%), and clindamycin in 24 cases (3.5%). Combinations of the above antibiotics were used in 207 cases (30.9%).

**Table 2:** Group compositions of the different treatment groups

	All	Single-shot	48 h	> 72 h
Mean age (in years)				
< 18 years (*n*) ≥ 18 years (*n*)	42.3 172 498	54.2 7 60	47.8 27 131	39.3 138 307
Sex (*n*)				
Male Female	305 365	23 44	60 98	222 223
Immunosuppressive pre-existing conditions/coagulopahtie (*n*)	58	8	18	32
smoking (*n*)	96	8	26	62
Regular alcohol consumption (*n*)	30	2	11	17
Length of hospital stay (in days)	6.37	6.37	6.29	6.46
Infection (*n*)				
Major Minor	64 12 52	8 3 5	17 0 17	39 9 30
All (*n*)	670	67	158	445

Overall, 64 of the 670 patients (9.5%) showed wound infection within the first 28 postoperative days after CI. Major infection was noted in 11 patients (1.6%). (Table 3) One of these patients (0.1%) needed explantation because of recurrent infections in the further course. Minor infections occurred in 53 patients (8%). Overall, 8 (11.9%) infections occurred in the single shot group, 17 (10.6%) in the 48 h group, and 39 (8.8%) in the 72 h group. Major infections occurred in the single shot group in 3 patients. No major infections were described in the 48 h group. In the 72 h group, 8 major infections occurred.

**Table 3:** Major complications

No.	Sex	Age	Predisposing conditions	Group of AB-prophylaxis	Time after surgery (days)	Clinical presentation of infection	Therapy/type and duration of AB	Bacterium responsible
1	w	1	None	> 3 days	5	Postoperative fever	AB (Cefuroxim) 3 days i.v., 5 days p.o.	n.d.
2	w	3	1	> 3 days	7	SSI	AB (Cephazolin) 8 days i.v. 4 days p.o./SR	n.d.
3	w	78	1.2	SS	7	SSI	i.v. AB (Cefuroxim) 4 days i.v., 5 days p.o.	n.d.
4	w	81	None	SS	2	SSI	AB (Cefuroxim) 3 days i.v.	n.d.
5	w	70	None	SS	20	Wound dehiscence	AB (Cephazolin) 5 days i.v., 7 days p.o./SR	*Staphylococcus aureus*, n.r.
6	w	38	None	> 3 days	3	Seroma	AB (Cefuroxim) 5 days i.v.	n.d.
7	m	62	3	> 3 days	4	hematoma	AB (Cefuroxim) 7d i.v.,4 d p.o.	n.d.
8	w	47	None	> 3 days	2	SSI	AB (Cefuroxim) 3 days i.v.	n.d.
9	w	78	None	> 3 days	9	wound dehiscence	AB (Cefuroxim) 4 d i.v.	n.d.
10	m	46	None	> 3 days	6	wound dehiscence	AB (Cefuroxim), 10 d i.v./SR	n.d.
11	m	68	3	> 3 days	20	SSI	AB (Piperacillin/Sulbactam) 7 days i.v., (1 month later: AB, SR, 4 month later: AB, explantation)	*Pseudomonas aeruginosa*, resistant to Levofloxacin and Ciprofloxacin

Predisposing conditions: 1: history of chronic otitis media 2: preexisting conditions or medication affecting the immune system 3: preexisting conditions or medication affecting the blood clotting

*SS* single shot, *SSI* surgical side infection, *AB* type of AB therapy-administration, *SR* surgical revision, *n.d.* not detected, *n.r.* no resistances to antibiotics, *p.o.* per os, *i.v.* intravenous

There was a slight, statistically non-significant increase in the risk of infection between the 72 h group and those who received single shot antibiosis (OR 1.41 [95% CI 0.63–3.17], *p* = 0.4) or 48 h prophylaxis (OR 1.26 [95%CI 0.69–2.29], *p* = 0.46). Using the Fisher’s exact test there was no statistically significant difference between the infection rate with a single dose at 11.9% (8/67), as well with a 48 h administration at 10.6% (8/67) compared to a 3-day dose at 8.8% (39/445) (*p* = 0.37, Cramer's *V* = 0.04).

**Fig. 1:**
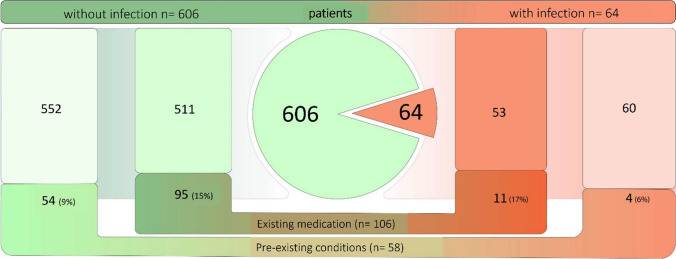
Distribution of patients with pre-existing conditions/pre-medication with possible influence on postoperative wound healing disorders in relation to the patient group with and without postoperative infections. From 670 Patients 64 patients suffered from postoperative wound infections. Of these, 11 (17%) patients had medication and 4 (6%) patients had preexisting conditions that may have had an influence on the postoperative infection risk. In the group of patients without postoperative infections (*n* = 606) 95 (15%) patients had such medication and 54 (9%) preexisting conditions

Patients with previous ear surgery (OR 1.06 [95% CI 0.56–2.01], *p* = 0.85), immunosuppressive history (OR 0.65 [95% CI 0.23–1.86], *p* = 0.42) or immunosuppressive medications (OR 0.81 [95% CI 0.1–6.36], *p* = 0.84) were not found to have a statistically significant cluster of infections compared to patients without these risk factors (Fig. 1). Similarly, the choice of antibiotic (amoxicillin /clavulanic acid or ampicillin/sulbactam, cefuroxime, cephazolin, clindamycin, or a combination of these agents) did not appear to have a relevant influence (*p* = 0.44).

In 6 cases of major infections in adults, there was mild redness/swelling in the area of the retro-auricular wound, which completely regressed under intravenous antibiotic treatment and there were no further complications in the further course. In 3 patients, wound revision and prolonged antibiotic treatment was performed. In 1 patient this could be done under local anesthesia, in 2 patients general anesthesia was necessary. The pathogens detected were *Staphylococcus aureus *in one case and *Pseudomonas aeruginosa *in the other case. In the patient with Pseudomonas, recurrent infections occurred in the further course, so that the CI had to be removed after approx. 4 months.

Focusing on the group of children (< 18 years) within our patient collective, postoperative infections occurred in a total of 16 patients (8.7%). Of these, two infections were major complications (1.1%). Both major complications occurred although antibiotic therapy had been administered for > 72 h. In a one-year-old patient, a new-onset unclear fever occurred on the 5th postoperative day after discharge, and the patient was readmitted to the hospital. There were no clinical signs of wound infection. A three-year-old patient developed a superinfected hematoma in the area of the implant site on the 7th postoperative day, so that surgical drainage as well as intravenous antibiotic therapy were necessary. The implant could be preserved.

Evidence for a statistically significant increase in the risk of infection in children compared to adults was not found in our data (OR 0.89 [95% CI 0.49–1.60], *p* = 0.69).

## Discussion

Aiming to evaluate the importance of perioperative antibiotic administration in CI surgery, to our knowledge our study represents the largest monocentric evaluation to date (*n* = 670). Like all other studies published on this topic, our work is retrospective in nature, which is known to have some drawbacks such as sampling bias and methodological limitations.

Studies with similarly high or higher case numbers always describe multicenter patient collectives [6]. In a multicenter setting different surgeon, surgical techniques, decision criteria, preoperative and postoperative protocols represent a multitude of possible confounders. For example, preoperative preparation of the skin and the surgical field as well as the length and shape of the skin incision are suspected to have an influence on the postoperative wound infection rate [7, 8]. In some centers the implant is soaked in an antibiotic solution before insertion [7, 9]. Some surgeons create a bony bed for the implant to avoid dislocation, others secure it simply in a subperiosteal pocket [10]. The management of patients who are at increased risk of infection due to a history of chronic otitis media also varies from center to center [11].The patient groups we described were all treated at the same hospital according to an established protocol. Thus, other influencing factors should be minimalized respectively are the same in all groups and the influence of different antibiotic regimens could be analyzed without major confounders.

Our data show an overall postoperative wound infection rate of 9.5% with a risk of major complications of 1.7%. These data are consistent with what is reported in the mainstream literature. Here, infection rates ranging from 1 to 13% are reported, but these data are often inconsistent regarding the observation period and the distinction between minor and major complications. A recent review on the role of perioperative antibiotic prophylaxis includes 6 studies [1]. Compared with infection rates described here, abnormalities in wound healing were documented relatively frequently in our clinic (Table 4). This could be explained by the close postoperative control intervals in our protocol. However, these findings rarely led to major complications. Explantations could be prevented with only one exception, which happened beyond the defined observation period after recurrent infections a.e. due to formation of a biofilm. This represents a very low explantation rate compared to the international literature. Neither meningitis nor intracerebral complications occurred. Thus, close clinical monitoring and early treatment may be a favorable strategy to avoid serious events such as explantation.

**Table 4:** Comparison of infection rates reported in the literature

Study	Patients (*n* = )	Infections (*n* =)	Minor (*n* = /%)	Major (*N* = /%)	Explantations (*N* = %)	Time of observation (M)
Sayed-Hassan et al. [6]	1180	12	n.u.	12/1%	5/0.4%	12
Basavaraj et al. [16]	292	12	8/2%	4/1.4%	2/0.7%	1
Hirsch et al. [12]	95	3	3/3%	0	0	
Almosnino et al. [9]	188	0	n.u.	0	0	1
Garcia-Valdecasas et al. [13]	196	9	0	9/4.6%	9/4.6%	4
El-Saied et al. [14]	130	6	6/4.6%	0	0	1
Our data	670	64	53/8%	11/1.6%	0	1

### Duration of antibiotic administration

There was no evidence for prolonged antibiotic administration providing any benefit compared with an intraoperative single shot alone. A similar conclusion was drawn in the study published in 2018 by Almosino et al. [9]. Also here, there was no difference in wound infection rates between patients who received an intraoperative single shot only and those who received an additional five days of antibiotic therapy. Postoperative infections were not documented in any of the patients. However, the small number of cases in this study (*n* = 188) limits the validity of this statement. Basavaraj et al. concluded that patients who received longer-term antibiotic therapy had a higher infection rate than patients who received only an intraoperative single shot [15].

In contrast to our study, a large multicenter study from France [6] concluded that longer-term perioperative antibiotics lead to fewer infections compared with single shot, especially in children. With 1180 patients, Sayed-Hassen et al. describe the largest patient collective to date. With an overall infection rate of 1.0%, 8 patients from the "prophylaxis group" (< 48 h of antibiosis) and 4 patients who received longer-term antibiosis > 48 h developed an infection. However, in contrast to our evaluation, only major infections were recorded here. The observation period was 1 year after implantation according to the definition of surgical site infection by the French National Authority of Health. Many of the infections occurred with a delay within a period of > 90 days up to 12 months. Thus, these no longer correspond to the definition of postoperative wound infections that is valid in Germany according to the "center of disease control" (CDC; 2017). Therefore, a surgical site infection must occur within the first 30 days (or 90 days if an implant is present) after surgery. Type and duration of antibiotic administration, perioperative protocols, and infection rates varied among the 8 involved centers. Children had a fourfold increased risk of infection compared with adults. Three times as many children in the prophylaxis group had an infection compared with the therapy group. Therefore, the authors generally recommend prolonged antibiotic therapy (> 48 h) in children. In our data, children did not show an increased risk of developing an infection compared with adults, nor was there any evidence of a benefit of prolonged antibiotic administration. However, the time frame for postoperative infections was set tighter according to the CDC. When restricted to 90 days postoperatively, the results of Sayed-Hassen et al. are comparable to ours. Therefore, we consider a single shot of antibiotics to be sufficient also in children and thus agree with the study by Saied et al. Here, 130 children did not show an increased risk of infection with shorter antibiotic prophylaxis (24 h i.v. versus 24 h i.v. plus 1 week oral administration). Children are most likely to be at higher risk for otogenic infections in the longer term. This is why close clinical monitoring is useful, especially if there are signs of upper respiratory tract infections.

In addition to the studies already mentioned, a recent review [1] describes two further studies that have dealt with the role of perioperative antibiotic prophylaxis. Hirsch et al. judged single shot prophylaxis alone to be sufficient for an infection rate of 1% in 94 patients, but without opposing this to a control group. Garcia-Valdecasas et al. describe a clear benefit of an additional long-term antibiotic (clarithromycin for 6 weeks) versus a preoperative single shot of ceftriaxone. Due to the heterogeneity of the above-mentioned studies and the lack of prospective, randomized and controlled studies, the authors of the review conclude that there is insufficient evidence to form a consensus or recommendation based on the available data.

### Pre-existing conditions/pre-surgeries

Patient comorbidities are other possible risk factors for postoperative infections mentioned in the literature [16]. We could not confirm this association in our patient population neither for immunosuppressive comorbidities such as diabetes mellitus, nor for nicotine or regular alcohol consumption. Chronic middle ear infections and previous operations, such as the presence of radical cavities for example, are also considered to be factors favoring infection [17, 18]. In our data, we see no correlation probably because in these patients, the CI was inserted in a two staged procedure after the ear had been rehabilitated (either by perfomring a tympanoplasty or a subtotal petrosectomy and obliteration of the auditory canal and middle ear).

### Type of antibiotics

In accordance to the literature, the antibiotics cefuroxime, clindamycin and amoxicillin/clavulanic acid or ampicillin/sulbactam were used, since their spectrum of activity covers the common pathogens of the skin and middle ear. In two cases of major complications, we could detect Staphylococcus aureus and *Pseudomonas aeruginosa*. The patient whose swab showed *Pseudomonas aeruginosa *colonization developed recurrent infections followed by explantation in the further course. This is consistent with other published data, which indicate that infections with *Pseudomonas aeruginosa* were felt to be more sinister and more often lead to explantation [5]. As a consequence of the severe course of these kind of infections one should be aware of the risk and perform early microbiologic testing in order to treat the condition adequately. However, due to the very rare occurrence of such severe complications (1/700 in our cohort), it does not seem to be reasonable to adapt antibiotic prophylaxis to this rare pathogen in a standardized manner. The risk of inducing resistance and increasing side effects would by far outweigh the possible benefits.

## Summary

In our study, there was no statistically significant evidence for an increased risk of postoperative infection by reducing perioperative antibiotic administration to an intraoperative single shot. The sparing use of antibiotics seems reasonable. Further studies with randomized, controlled, and prospective study protocols are needed to form an evidence-based recommendation. Due to the low incidence of postoperative infections, a multicenter survey with a high number of cases would also be desirable in prospective protocols.

## Data Availability

Not available.

## References

[1] Kajal S, Mishra A, Gupta P et al (2022) Duration of antibiotic prophylaxis for cochlear implantation: a systematic review. J Int Adv Otol 18:269–275. 10.5152/iao.2022.21454 35608498 10.5152/iao.2022.21454 PMC10682799

[2] Deutsche Gesellschaf für Hals- Nasen- Ohrenheilkunde, Kopf- und Halschirurgie e.v., S2k-Leitlinie Cochlea-Implantat Versorgung, 3.0, 31.10.2020

[3] Empfehlung der Kommission für Krankenhaushygiene und Infektionsprävention (KRINKO) beim Robert Koch-Institut, Prävention postoperativer Wundinfektionen, Bundesgesundheitsblatt Gesundheitsforschung Gesundheitsschutz 61:448–473. 10.1007/s00103-018-2706-2 10.1007/s00103-018-2706-2 29589090

[4] Cohen NL, Hirsch BE (2010) Current status of bacterial meningitis after cochlear implantation. Otol Neurotol 31:1325–1328. 10.1097/MAO.0b013e3181f2ed06 20818287 10.1097/MAO.0b013e3181f2ed06

[5] Kabelka Z, Groh D, Katra R et al (2010) Bacterial infection complications in children with cochlear implants in the Czech Republic. Int J Pediatr Otorhinolaryngol 74:499–502. 10.1016/j.ijporl.2010.02.007 20394849 10.1016/j.ijporl.2010.02.007

[6] Sayed-Hassan A, Hermann R, Chidiac F et al (2019) Association of the duration of antibiotic therapy with major surgical site infection in cochlear implantation. JAMA Otolaryngol Head Neck Surg 145:14–20. 10.1001/jamaoto.2018.1998 30325991 10.1001/jamaoto.2018.1998 PMC6439807

[7] Clark GM, Pyman BC, Pavillard RE (1980) A protocol for the prevention of infection in cochlear implant surgery. J Laryngol Otol 94:1377–1386. 10.1017/s0022215100090204 6893717 10.1017/s0022215100090204

[8] Gawęcki W, Karlik M, Borucki Ł et al (2016) Skin flap complications after cochlear implantations. Eur Arch Otorhinolaryngol 273:4175–4183. 10.1007/s00405-016-4107-1 27245752 10.1007/s00405-016-4107-1 PMC5104790

[9] Almosnino G, Zeitler DM, Schwartz SR (2018) Postoperative antibiotics following cochlear implantation: are they necessary? Ann Otol Rhinol Laryngol 127:266–269. 10.1177/0003489418758101 29429348 10.1177/0003489418758101

[10] Vijendren A, Borsetto D, Barker EJ et al (2019) A systematic review on prevention and management of wound infections from cochlear implantation. Clin Otolaryngol 44:1059–1070. 10.1111/coa.13444 31561283 10.1111/coa.13444

[11] Davids T, Ramsden JD, Gordon KA et al (2009) Soft tissue complications after small incision pediatric cochlear implantation. Laryngoscope 119:980–983. 10.1002/lary.20204 19334044 10.1002/lary.20204

[12] Hirsch BE, Blikas A, Whitaker M (2007) Antibiotic prophylaxis in cochlear implant surgery. Laryngoscope. 117(5):8664–867 10.1097/MLG.0b013e318033c2f9 17473684

[13] Garcia-Valdecasas J, Jiménez-Moleon JJ, Sainz M, Fornieles C, Ballesteros JM (2009) Prophylactic effect of clarithromycin in skin flap complications in cochlear implants surgery. Laryngoscope 119(10):2032–2036 19688847 10.1002/lary.20603

[14] El-Saied S, Joshua BZ, Abu Tailakh M et al (2018) Early postoperative fever in paediatric patients undergoing cochlear implant surgery. Clin Otolaryngol. 43(1):358–388 10.1111/coa.13009 29044978

[15] Basavaraj S, Najaraj S, Shanks M et al (2004) Short-term versus long-term antibiotic prophylaxis in cochlear implant surgery. Otol Neurotol 25:720–722. 10.1097/00129492-200409000-00012 15354001 10.1097/00129492-200409000-00012

[16] Gluth MB, Singh R, Atlas MD (2011) Prevention and management of cochlear implant infections. Cochlear Implants Int 12:223–227. 10.1179/146701011X12950038111576 22251810 10.1179/146701011X12950038111576

[17] Incesulu A, Kocaturk S, Vural M (2004) Cochlear implantation in chronic otitis media. J Laryngol Otol 118:3–7. 10.1258/002221504322731538 14979963 10.1258/002221504322731538

[18] Hunter JB, O’Connell BP, Wanna GB (2016) Systematic review and meta-analysis of surgical complications following cochlear implantation in canal wall down mastoid cavities. Otolaryngol Head Neck Surg 155:555–563. 10.1177/0194599816651239 27221577 10.1177/0194599816651239

